# Impact of dry rice grain packing density and time on the performance of two stage dehumidifier

**DOI:** 10.1038/s41598-025-00339-1

**Published:** 2025-05-15

**Authors:** Shiva Kumar, Sampath Suranjan Salins, Uday Krishna Kuroor, Sloane Winslow D’Souza, Anupam Bose

**Affiliations:** 1 Department of Mechanical and Industrial Engineering, Manipal Institute of Technology, Manipal Academy of Higher Education, Manipal, 576104 India; 2 Manipal Academy of Higher Education, PO 345050, Dubai Campus, UAE

**Keywords:** Solid desiccant, Rice grains, Heat and mass transfer, Multistage dehumidifier, Transient, Energy science and technology, Engineering

## Abstract

This study presents the design and development of a multistage solid desiccant dehumidifier using rice grains as the desiccant material. The rice packing densities varied between 1100 and 4390 kg/m^3^, and experiments were conducted to assess performance over different time intervals. Results indicated that system performance improved with increasing packing density up to 3290 kg/m^3^, after which a decline was observed. Additionally, performance improved with time, stabilizing after approximately 15 min of operation. The system achieved a maximum moisture removal rate of 0.18 g/s, a dehumidification efficiency of 73.57%, a coefficient of performance (COP) of 4.35, and a mass transfer coefficient of 20.40 kg/m^2^·s. The highest dehumidification efficiency was recorded in the first stage, with a notable reduction in the second stage. Velocity reductions from top to bottom for packing densities of 1100 kg/m^3^, 2190 kg/m^3^, 3290 kg/m^3^, and 4390 kg/m^3^ were 25%, 38.88%, 51.51%, and 66.66%, respectively. While the exhaust air met ASHRAE thermal comfort standards, an increased pressure drop was observed, with a maximum value of 180 Pa recorded.

## Introduction

As communities grow, the demand for improved living standards rises, especially in hot & humid climates. Thermal comfort is achieved by reducing moisture and lowering air temperature, while industries also require low humidity conditions^1,2^. Traditional refrigeration systems use compressors to remove moisture through condensation by cooling the air lower than its dew point. However, desiccant cooling systems provides an effective substitute to vapor compression refrigeration (VCR), reducing energy consumption, maintenance, and system size^3,4^. In some cases, a hybrid system combining solid desiccant dehumidifiers with VCR enhances efficiency by supporting both cooling and moisture removal. Solid desiccants are naturally available elements that adsorb vapor of water due to partial vapor pressure difference between desiccant & surrounding air^5,6^. They offer benefits such as low carryover, easy maintenance, compact size, and low-temperature regeneration. Commonly used solid desiccants are silica gel (Si O_2_), zeolite & alumina^7^. Several factors affect the performance of solid desiccant dehumidifiers, including the type of packing, flow configuration, regeneration method, packing position or movement, flow velocity, and vapor pressure difference between desiccant & circulated air^8^. Dehumidification in stages can improve overall efficiency. Other important parameters that impact performance include the duration and density of the packing. Achieving the optimal packing density is essential, as deviations can result in a significant drop in exit velocity & rise in pressure drop.

Several researchers have investigated solid desiccant dehumidifiers using different materials for the purpose of dehumidification. Aziz et al.^9^ studied four distinct packing materials, including Metal–Organic Frameworks (CPO27(Ni), MIL100(Fe), MIL-101(Cr), Aluminium Fumarate), as well as silica gel, in a stationary desiccant heat exchanger coated with these frameworks. Their findings revealed that the Aluminium Fumarate coating delivered superior performance, with the moisture removal rate increasing as the humidity rose and air velocity decreased. Chaudhary et al.^10^ analysed a silica gel dehumidifier, validating its thermodynamic model under different operating conditions. The results indicated that an optimal wheel rotation speed of 15 to 17 rph achieved an effectiveness of 0.43, with a regeneration temperature of 80ºC. Dadi and Jani et al.^11^ conducted experiments with a constant air flow rate of 0.07 kg/s & fixed wheel speed of 20 revolutions per hour. The results demonstrated that unit performed effectively in hot & humid climates, reducing the specific humidity by 59.01%. The study concluded that system performance was significantly influenced by outdoor conditions. Hussain et al.^12^ developed a silica gel-based desiccant air conditioning system, achieving an adsorption capacity of 4.88 g/kg. Energy analysis revealed that the system consumed 0.64 kW of latent heat and 1.16 kW of sensible heat. Kumar et al.^13^ studied solid composite desiccant blend made from coconut shell-based activated carbon, which was used to coat aluminium plates. Heat exchanger was cooled with cerium oxide nanofluid, and dehumidification occurred by passing air over the plates. Experiments varied the air velocity, rate of water flow & the concentration of the nano particles. The results showed that dehumidification performance improved with increased air velocity, rate of water flow, & concentration of desiccants. Li et al.^14^ assessed the performance of silica gel & sodium polyacrylate desiccants coated onto finned tube heat exchangers. Both single & two-stage desiccant-coated heat exchangers (DCHEs) were studied, with two-stage DCHEs featuring a dense coating showing superior performance. Additionally, sodium polyacrylate DCHEs demonstrated 1–1.3 times higher COP compared to silica gel DCHEs at high humidity levels. Aleem et al.^15^ developed a lab-scale solid desiccant dehumidifier and tested two desiccants—silica gel and hydrophilic polymeric sorbents—to evaluate their dehumidification capacity. The hydrophilic polymeric sorbents demonstrated superior performance. Cheng et al.^16^ studied various desiccants and found that lithium bromide (Li Br) provided a higher COP compared to magnesium chloride (MgCl_2_) and calcium chloride (CaCl_2_) desiccants. Zhi and Yang^5^ employed a variable frequency fan to control the air flow rate, along with a heating element for regeneration. The system achieved a high dehumidification efficiency of 85% and improved energy efficiency by 8.1% compared to traditional dehumidifiers. Tu and Hwang et al.^17^ conducted similar research utilizing heat pump energy for regeneration. They improved dehumidifier performance by automating the process with a switching time mechanism, which resulted in an increased COP. The optimal switching time was found to be between 3 and 5 min. Xue et al.^18^ proposed a suspension bridge hybrid dehumidification system (HDS) designed to protect against corrosion. The HDS unit harnesses energy from the bridge to achieve a specific moisture extraction rate (SMER) in hot & humid climates. The results showed that HDS outperformed the traditional desiccant wheel dehumidification system. Ge et al.^19^ studied Al-based Metal–Organic Frameworks (MOFs) for dehumidification in desiccant-coated heat exchangers (DCHE), focusing on MIL-96 & MIL-100, synthesized through aluminium dissolution. Their findings revealed that the MOFs exhibited a high dehumidification capacity of 0.35 kg/kg at 70% relative humidity. The MOF-coated heat exchanger (MCHE) outperformed silica gel-coated heat exchangers (SCHE) by a factor of 2 to 3 and demonstrated adaptability to various climatic conditions.

Some researchers worked on the multistage dehumidification system. Naik et al.^20^ studied a solid desiccant dehumidification unit, examining the influence of inlet velocity, humidity & temperature. Their findings indicated that a two-stage solid desiccant dehumidifier was particularly effective for deep drying processes and performed well in humid climatic conditions. Yang et al.^21^ conducted experiments on a three-stage solid desiccant adsorption unit and assessed its performance. They found that the first stage of dehumidification was critical to the overall performance. Salins et al.^22^ worked on multistage dehumidification system, conducting experiments with varying the velocity of air, cam shaft speed & relative humidity (RH). The multistage dehumidifier outperformed the single-stage unit, achieving a maximum dehumidification efficiency of 76.2% & moisture removal rate of 7.54 g/s. Tu et al.^23^ developed a multistage dehumidifier that utilized exhaust heat from heat pump for regeneration. They investigated the effects of inlet air conditions and wheel speeds, achieving a coefficient of performance (COP) of 5.5. The two-stage unit notably improved dehumidification performance. Al Ezzi et al.^24^ investigated the adsorption and regeneration performance of zeolite desiccants for indoor dehumidification. At a Reynolds number of 1773 and 99% relative humidity, complete adsorption was achieved in 37 min, while regeneration at 100 °C was completed in 66 min. Sunhor et al.^25^ investigated a crossflow heat exchanger coated with aluminophosphate zeolite for desiccant dehumidification using hot water regeneration. Their findings indicated that the regeneration temperature should exceed 65 °C for effective performance. Yao et al.^26^ developed an experimental silica gel dehumidifier utilizing ultrasonic regeneration. Results showed that higher regeneration temperatures enhanced both the moisture removal rate and coefficient of performance. Additionally, while increased humidity improved the moisture removal rate, it led to a decrease in the coefficient of performance. Liu et al.^27^ studied desiccant-coated heat exchangers using polymer desiccants. Their results indicated that the dual-stage unit achieved a higher moisture removal rate and coefficient of performance compared to single-stage systems.

After conducting an extensive literature review, it was found that many researchers have worked on Metal–Organic Frameworks (such as CPO27(Ni), MIL100(Fe), MIL-101(Cr), & Aluminium Fumarate) for dehumidification. Desiccants like silica gel, sodium polyacrylate, lithium bromide (Li Br), magnesium chloride (Mg Cl₂), and calcium chloride (Ca Cl₂) are commonly used. Dehumidification systems employing desiccant wheels with variable speeds are widely utilized, alongside static-type systems. In some cases, multistage dehumidifiers are also employed, and it has been found that they enhance overall performance. Although extensive research has been conducted on dehumidification, particularly with various solid desiccants, the use of rice grains as a dehumidifying agent remains limited because of its short life span and limited moisture absorption.

Rice can be used as a dehumidification agent in some special applications, such as to reduce moisture in containers storing grains, spices, or seeds, to dry small electronic devices like smartphones or cameras exposed to moisture where the use of chemical-based solid desiccants is not advisable. The performance studies such as Moisture removal rate (MRR), efficiency & regeneration capacity etc. for rice grains have not been widely explored. Most studies have concentrated on single-stage dehumidifiers, while the investigation of multistage dehumidifiers and the changes in dehumidification performance across different stages represents a novel approach. Additionally, there is limited research on the impact of packing density variations. To address these gaps, a two-stage dehumidifier test rig has been developed, and dry rice grains with varying densities are employed to assess dehumidification performance metrics such as MRR, dehumidification efficiency, COP, mass transfer coefficient, specific humidity change & temperature variation.

## Principle of two stage dehumidification along with the performance parameters

**Fig. 1:**
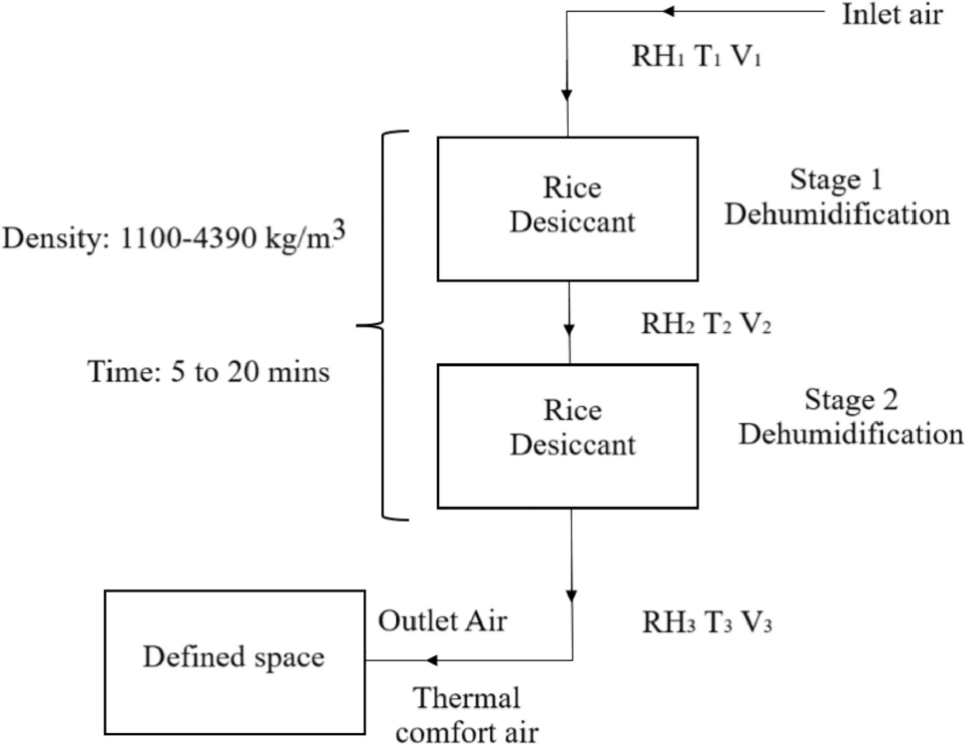
Two stage rice desiccant dehumidification.

Figure 1 illustrates the two-stage rice desiccant dehumidifier, where air is passed through two stages of varying desiccant densities. At the inlet, the temperature (T_1_), relative humidity (RH_1_), and velocity (V_1_) of the air are recorded, with these parameters changing as air moves through every stage. The dehumidified air is then directed into a defined space, where conditions are maintained within thermal comfort standards. Performance parameters are determined based on input & output values, which are defined & explained by Eqs. (1) to (4). These parameters are analysed to assess the system’s performance, considering the variations in rice desiccant density and time.

Moisture removal rate (MRR) is defined as a product of change in specific humidity and air flow rate. Humid air from the atmosphere is passed through two stages of the dehumidifier, which contain rice grains as the desiccant material. The moisture adsorbed or removed by the desiccant is calculated using Eq. (1). W_1_ and W_3_ represent the humidity ratios at inlet & outlet, respectively, while $\dot{{m}_{a}}$ and $\dot{{m}_{w}}$ denote the air flow rate & rate of condensation or moisture removal rate^28^.

1 $$\dot{{m}_{w}}=\left({W}_{1}-{W}_{3}\right)\dot{{ m}_{a}}$$

The coefficient of performance (COP) represents energy efficiency of the unit. It is calculated as the ratio of the heating effect (HE) to power consumed by the blower, as expressed in Eq. (2). In this context, h_3_ & h_1_ refer to enthalpies at the outlet & inlet, respectively, while $\dot{{W}_{B}}$ represents the power consumed by blower^28^.

2 $$COP=\frac{\dot{Q}}{\dot{W}}=\frac{\dot{{m}_{a}}({h}_{3}-{h}_{1})}{ \dot{{W}_{B}}}$$

The efficiency of dehumidification is defined as the ratio of difference in specific humidities between the inlet & equilibrium specific humidities. This is expressed in Eq. (3). W_1_, W_2_, and W_eq_ represent the inlet, outlet, and equilibrium specific humidities, respectively^28^.

3 $${\eta }_{d}=\frac{{W}_{1}-{W}_{3}}{{W}_{1}-{W}_{eq}}$$

The mass transfer coefficient, as defined by Eq. (4), describes the transfer of mass occurring between two phases: air & solid desiccant. It is defined as the ratio of condensation rate to the product of the area & difference between the average & equilibrium specific humidities. W_av_ gives the average value of the specific humidities at the inlet and the outlet where as the W_eq_ is the equilibrium specific humidity for the particular dry bulb temperature and relative humidity. It is the point at which a material stops absorbing or releasing moisture to reach a balance with the humidity in its immediate environment^28^.

4 $$K=\frac{\dot{{ m}_{w}}}{A ({W}_{av}-{W}_{eq})}=\frac{\dot{{ m}_{w}}}{A ({\Delta W}_{3})}$$

## Construction and working

**Fig. 2:**
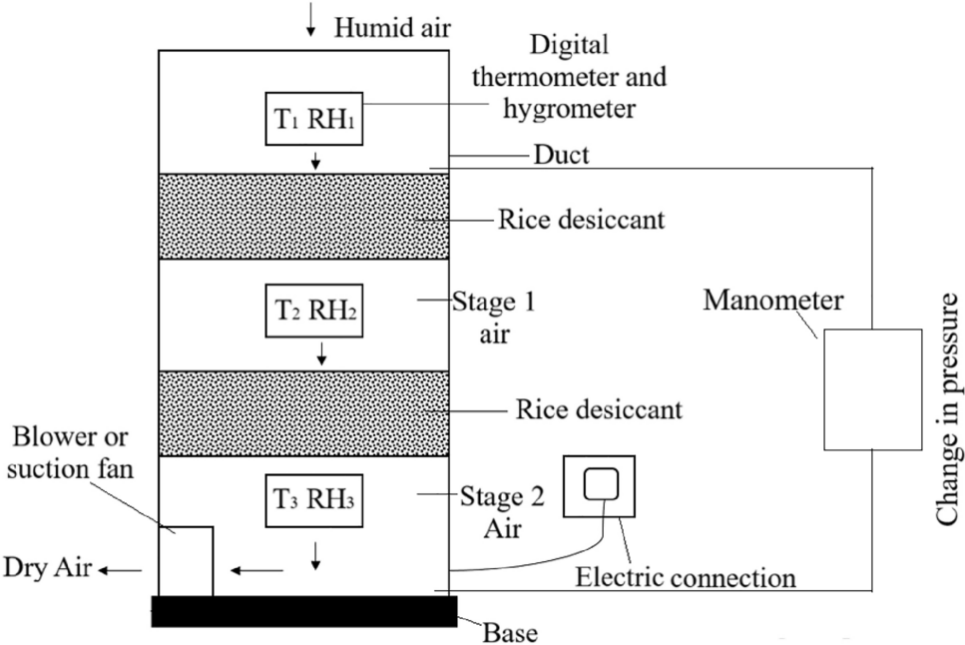
Schematic sketch of rice desiccant dehumidifier.

The system features a vertical column constructed from durable thermocol duct material covered by a thermal blanket serving as an insulator. A 0.1 HP blower is installed at the bottom of the column to generate suction, powered by electricity. The entire unit is supported on a base. Dry rice of varying densities is positioned between the steel wire mesh, located in the middle and bottom sections of the column. Rice desiccant has characteristics such as a specific heat capacity of 1.4 to 1.6 J/g·°C, thermal conductivity between 0.2 and 0.3 W/m·K, and 40–60% porosity. The rice grains are approximately 5–7 mm in length and 2 mm in width, providing a relatively lower surface area in comparison to other desiccants. Figure 2 illustrates the schematic design of the vertical-axis two-stage dehumidifier.

The densities were calculated by dividing the mass of the desiccant with volume—100, 200, 300, and 400 g—by a fixed volume of 13.5 cm × 13.5 cm × 0.5 cm. The range of masses was selected to represent the minimum to maximum capacity of the dehumidification volume. Four different desiccant densities—1100, 2190, 3290, and 4390 kg/m^3^—were obtained and tested, based on the volume of the desiccant chamber. The range of masses was selected to represent the minimum to maximum capacity of the dehumidification volume.

The pressure drop in a dehumidifier column is measured by recording air pressure at various points, such as the inlet and outlet, using pressure manometer. The difference in pressure readings reflects the pressure drop, which helps assess airflow efficiency, moisture removal, and system performance, often correlating with changes in airflow rates and resistance due to the packing material and moisture removal process. Figure 2 shows the manometer used to measure the pressure.

**Fig. 3:**
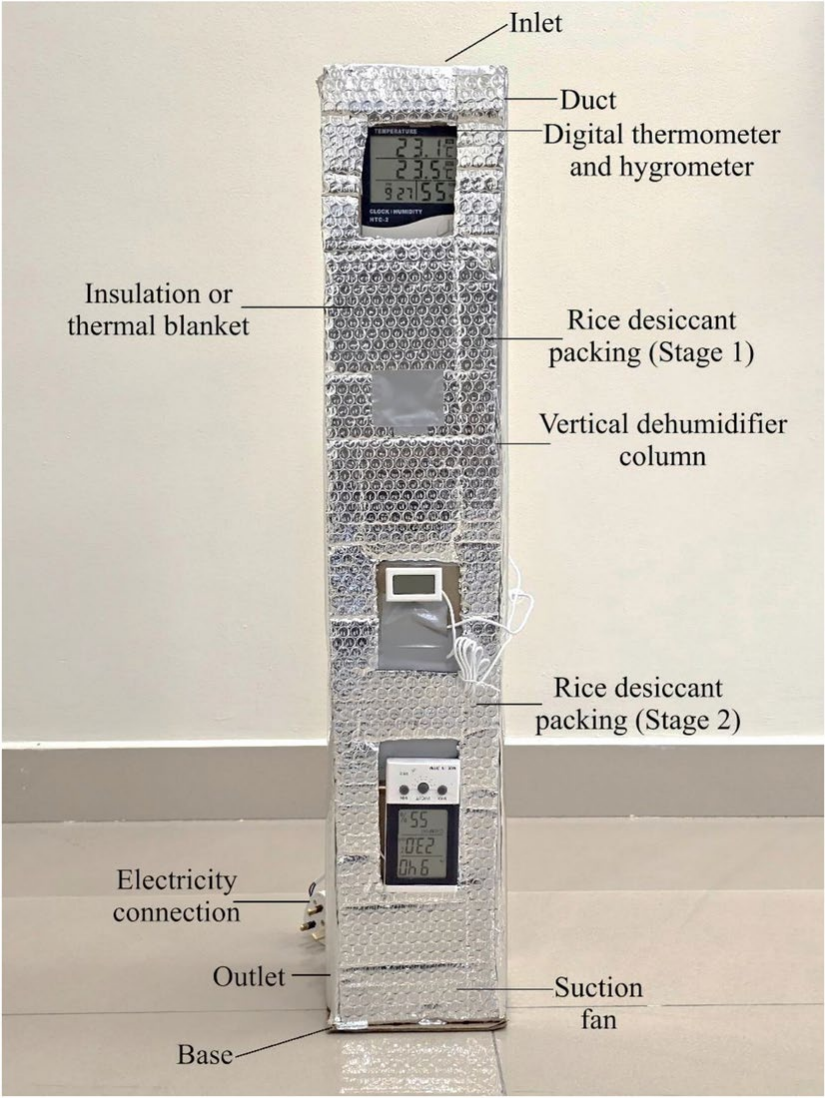
Constructed model of a rice desiccant dehumidifier.

**Fig. 4:**
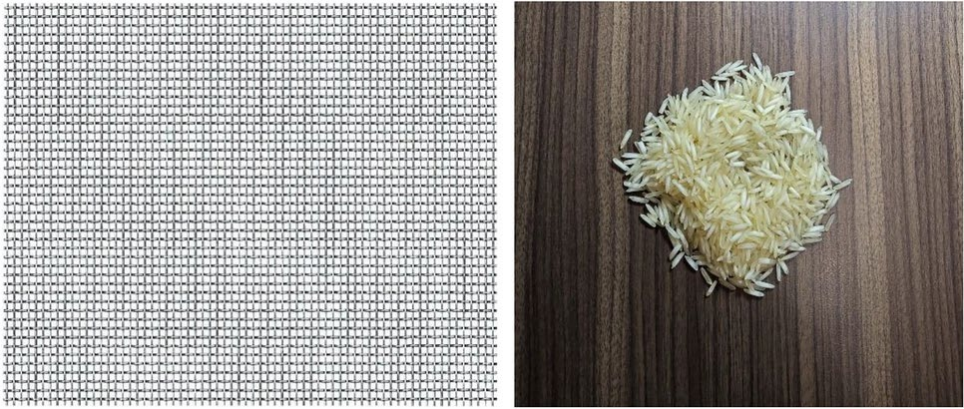
Stainless steel wire mesh and rice desiccant.

The blower is activated, causing the humid air to enter through the inlet and pass through two stages of dehumidification. As humid air interacts with rice desiccant, a vapor pressure difference between air & desiccant facilitates the removal of moisture, resulting in dry air being expelled. During this process, latent heat is converted into sensible heat, which raises temperature of exiting air. The dry air is then directed to designated space to maintain the desired dryness. The conditions of the inlet, intermediate, and outlet air are recorded, and using the psychrometric process, all thermal properties of the air are calculated. These parameters are substituted into Eqs. (1) to (4) to determine the output. Figure 3 depicts the fully assembled unit used for testing, while Fig. 4 shows the rice placed inside the mesh to support the dehumidification process.

### Instruments used in assessing the parameters in rice desiccant dehumidifier

Measuring instruments are utilized to obtain the condition of air at inlet, intermediate & outlet stages. A digital dry bulb thermometer is used to measure temperature with an accuracy ± 0.1 °C, resolution 0.1 °C, and a range of 0–80 °C. An anemometer is employed to measure air velocity, with range of 0 to 45 m/s, resolution of 0.1 m/s, & accuracy of ± 0.1 m/s. A hygrometer is used to measure RH of air, with range of 1–99%, a resolution of 0.1%, and an accuracy of ± 0.1%.

## Uncertainty analysis and experimental conditions

The root sum of squares (RSS) analysis method is used to determine the overall uncertainty of each performance parameter in the current rice desiccant dehumidifier unit. RSS combines multiple sources of uncertainty into a single value. This method is widely used in engineering, physics, and other fields that involve measurements. This method combines the independent uncertainties of multiple measurements to evaluate the total uncertainty of dependent variable. Let X represent independent variable, Y denote uncertainty intervals, G be the function, and Y_G_ the total uncertainty. The total uncertainty is calculated using Eq. (5)^28^.

5 $${{\text{Y}}_{G} = \left[{\left(\frac{\delta G}{\delta {X}_{1}} {Y}_{1}\right)}^{2}+ {\left(\frac{\delta G}{\delta {X}_{2}} {Y}_{2}\right)}^{2}+{\left(\frac{\delta G}{\delta {X}_{3}} {Y}_{3}\right)}^{2}+\dots \dots \dots \dots . +{\left(\frac{\delta G}{\delta {X}_{4}} {Y}_{n}\right)}^{2}\right]}^{0.5}$$

The uncertainty in the moisture removal rate, as shown in Eq. (1), is expressed using the RSS method.

6 $$\frac{{\partial \left( {\dot{m}_{W} } \right)}}{{\dot{m}_{W} }} = \sqrt {\left( {\frac{{\partial \dot{m}_{a} }}{{\dot{m}_{a} }}} \right)^{2} + \left( {\frac{{\partial \Delta {\text{W}}}}{{\Delta {\text{W}}}}} \right)^{2} }$$

The uncertainty associated with the evaluation of COP is given by Eq. (7).

7 $$\frac{\partial (COP)}{COP}=\sqrt{{\left(\frac{\partial \dot{Q}}{\dot{Q}}\right)}^{2}+\left(-\frac{\partial {\dot{W}}_{Blower}}{{\dot{W}}_{Blower}}\right)}$$

The dehumidification efficiency is provided by the equation below, and its uncertainty is given by Eq. (8).

$${\eta }_{d}=\frac{{W}_{1}-{W}_{3}}{{W}_{1}-{W}_{eq}}=\frac{{\Delta W}_{1}}{{\Delta W}_{2}}$$

8 $$\frac{\partial ({\eta }_{d})}{{\eta }_{d}}=\sqrt{{\left(\frac{\partial \Delta {\omega }_{1}}{\Delta {\omega }_{1}}\right)}^{2}+{\left(-\frac{\partial \Delta {\omega }_{2}}{\Delta {\omega }_{2}}\right)}^{2}}$$

The error in the mass transfer rate, as shown below, is described by Eq. (9).

$$K=\frac{\dot{{ m}_{w}}}{A ({W}_{av}-{W}_{eq})}=\frac{\dot{{ m}_{w}}}{A ({\Delta W}_{3})}$$

9 $$\frac{\partial (K)}{K}=\sqrt{ {\left(\frac{\partial (\dot{{ m}_{w}})}{\dot{{ m}_{w}}}\right)}^{2}+\left({\left(-\frac{\partial (\text{A})}{\text{A}}\right)}^{2}+{\left(-\frac{\partial (\Delta {\omega }_{3})}{\Delta {\omega }_{3}}\right)}^{2}\right)}$$

Change in the pressure is evaluated by using the equation. Let ρ be the density, kg/m^3^, g is acceleration due to gravity, m/s^2^ and z is the height of the fluid in the manometer.

$$P=\rho g\left({Z}_{2}-{Z}_{1}\right)=\rho g$$ ΔZ.

The error present while measuring the pressure us given by (10).

10 $$\frac{\partial (p)}{p}=\sqrt{\frac{\partial (\Delta \text{Z})}{\Delta \text{Z}}}$$

After conducting the error analysis, the total errors associated with measuring performance parameters of rice desiccant dehumidifier are evaluated using Eqs. (6) to (10) and presented in Table 1. Table 2 gives the experimental conditions associated with this study.

**Table 1:** Uncertainty present while measuring performance parameters of rice desiccant dehumidifier.

Sl.no	Variable	Percentage uncertainty
1	Moisture removal rate	2.01
2	Dehumidification efficiency	1.42
3	Coefficient of performance	2.58
4	Mass transfer coefficient	2.19
5	Change in the pressure	1.87

**Table 2:** Experimental conditions to determine the performance of rice desiccant dehumidification.

Sl. no	Packing material with its properties	Variations	Inlet and outlet measuring parameters	Performance parameters	Remarks
1	Dry rice packing material Specific heat 1.4 to 1.6 J/g·°C, conductivity ranging 0.2 and 0.3 W/m·K Porosity of 40–60%	Density of rice packing: 1100, 2190,3290 and 4390 kg/m^3^ Time:0 to 20 min Number of stages:2	Dry bulb temperature (DBT), RH, air velocity & pressure across pads	MRR, coefficient of performance, efficiency of dehumidification and mass transfer coefficient	Tests are conducted at constant air velocity of 5 m/s & the results are evaluated with respect to the first and second stage rice packing

### Experimental conditions

## Results and discussions

Dehumidification experiments are performed by changing the density of rice desiccant between 1100 and 4390 kg/m^3^, duration from 0 to 20 minutes, and number of stages. In the experiment above, all measurements were taken at specific time intervals—5, 10, 15, and 20 minutes—and the corresponding experimental values were recorded. Based on these values, all performance parameters were evaluated with respect to both Stage 1 and Stage 2 over time. The gradual increase in experimental duration was intended to minimize measurement errors and ensure the system reached steady-state conditions. Inlet & outlet parameters, including temperature, humidity & air velocities, are measured at various points along the vertical column of the duct. Using these measurements, performance parameters such as MRR, COP, dehumidification efficiency, and mass transfer coefficient are calculated and analysed in relation to the rice desiccant density and the experimental time. This section is divided into

5.1 Dehumidification performance variation with the density, time and stages

5.2. Thermal comfort

5.2.1. Psychrometric graph representing the state of air in stage 2 after dehumidification

Thermodynamic assumptions:

1. The system is assumed to operate under steady-state conditions, meaning temperature, pressure, and flow rates remain constant over time.
2. Air is treated as an ideal gas for simplicity in calculations.
3. Heat losses to the surroundings are considered negligible.

Mass balance assumptions:

1. The mass of air and water vapor is conserved throughout the system.
2. There are no chemical changes in the air or desiccant; only physical moisture absorption occurs.
3. The mass flow rate of air is constant throughout the system.
4. Moisture removal is attributed solely to the rice desiccant, with no external influences.
5. The mass of the rice desiccant remains constant during operation, assuming no physical loss or degradation.

### Dehumidification performance variation with the density, time and stages

#### Change in the temperature

**Fig. 5:**
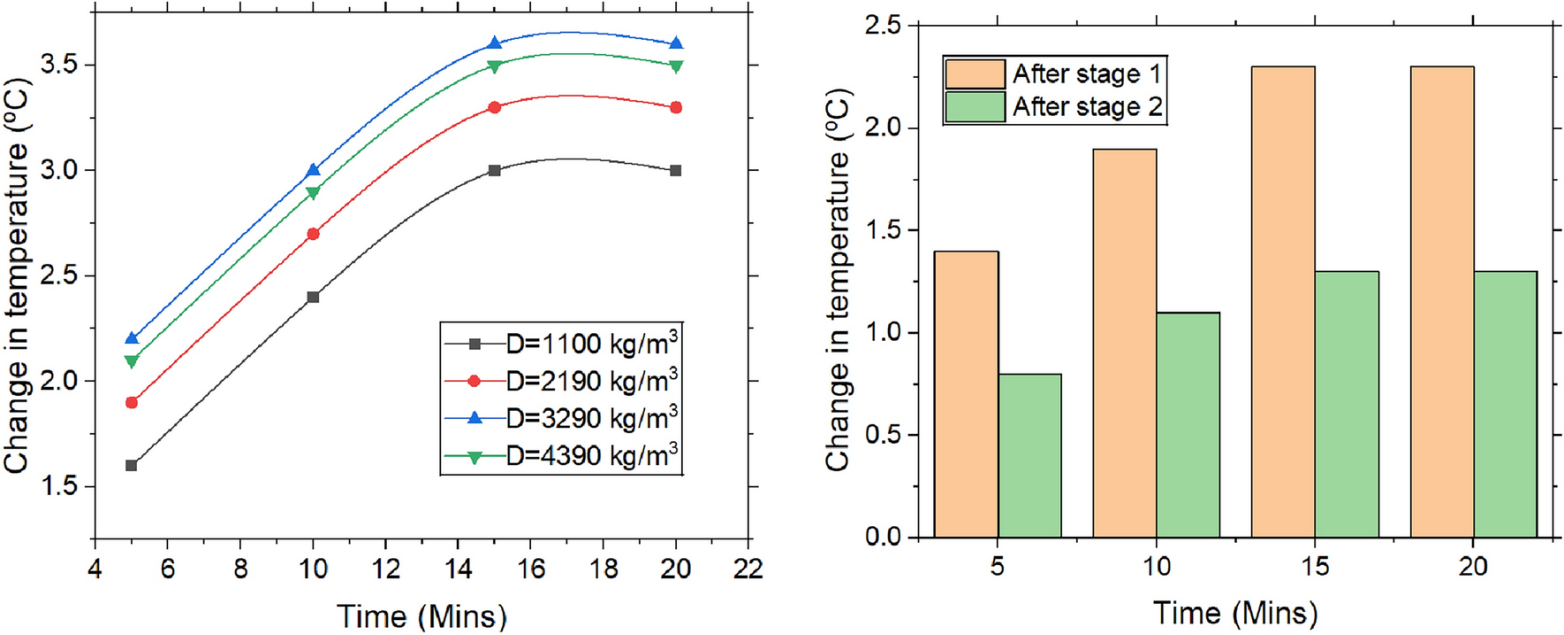
Change in temperature with the varying time, packing density and the stages.

Figure 5 depicts the variation in temperature change in relation to input parameters such as time, density, and the number of stages. As the dehumidification process advances, the desiccant progressively adsorbs more moisture. Being exothermic, this process generates heat, and as the desiccant becomes saturated, increased heat transfer to the surrounding air results in a rise in temperature. In the thermodynamic view point process involves the conversion of latent heat into sensible heat during moisture absorption. As humid air flows through the rice bed, the rice absorbs water vapor from the air. This phase change—from vapor to a bound or liquid state—releases the latent heat of condensation, which is not lost but transferred to the surrounding air and desiccant material. This transferred energy raises the air temperature, thereby increasing its sensible heat content. As a result, the air becomes drier (lower humidity) while its dry bulb temperature rises. This process demonstrates how latent heat is effectively transformed into sensible heat, in line with the first law of thermodynamics, which governs the conservation of energy.

This temperature increase continues as the experiment time extends. As time advances, heat disperses throughout the desiccant via conduction, thereby diminishing the temperature gradient. Upon reaching thermal equilibrium, rate of heat adsorption diminishes as temperature disparity between desiccant and its surroundings wanes. Ultimately, the temperature stabilizes when the absorption of heat aligns with its dissipation, sustaining a constant temperature over an extended duration. Density plays a pivotal role in moisture adsorption capacity of desiccant. A higher density enhances the desiccant’s mass, enabling it to adsorb more moisture and release additional heat during dehumidification. As density increases, greater heat is generated within the desiccant due to intensified molecular interactions between the desiccant and the air, further contributing to the temperature rise. Density of packing reaches 4390 kg/m^3^, increases collision frequency, allowing heat to spread more effectively. This results in a lower temperature rise in the air compared to a lower-density packing, where heat distribution is less efficient. The two-stage desiccant dehumidification process notably impacts the temperature increase. The moisture adsorbed in each stage progressively grows, leading to a cumulative heat buildup. It was observed that the majority of heat is generated in the first stage, causing the temperature to rise by 2.3 °C, which eventually decreases to 1.3 °C. With the time increase from 0 to 15 min, the temperature change escalated by 63.63%.

#### Change in the specific humidity

**Fig. 6:**
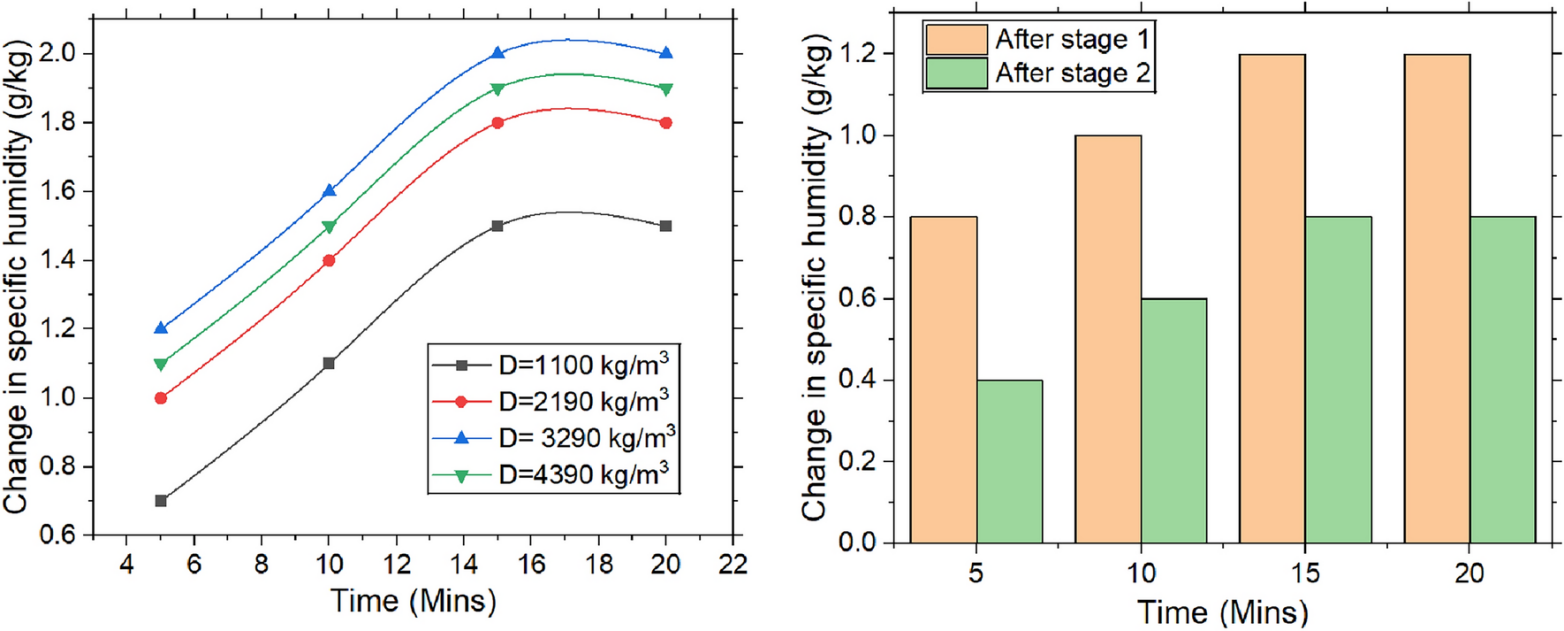
Change in specific humidity with the varying time, packing density and the stages.

Figure 6 illustrates the variation in specific humidity over time, which is a function of packing density and number of stages. The inlet relative humidity is maintained at 55%, and as air passes through rice desiccant, relative humidity decreases, leading to an increase in specific humidity change. At the molecular level, water molecules interact with the desiccant surface, where they are attracted by Van der Waals forces & hydrogen bonding. This adsorption reduces the amount of moisture exiting the system. It is observed that as time progresses, the desiccant has more opportunity to adsorb moisture, resulting in a greater change in specific humidity. Initially, the air has higher humidity because the rice desiccant is less saturated and more readily available for moisture adsorption. However, as time increases, the desiccant’s adsorption capacity decreases, leading to a reduction in water vapor concentration in air and a corresponding increase in specific humidity change.

The outlet specific humidity increased with packing density, as the desiccant absorbed moisture more effectively. Moisture adsorption rose with packing density up to 3290 kg/m^3^, beyond which the adsorption capacity significantly decreased. Water molecules form hydrogen bonds with active sites on the desiccant surface. In materials like rice packing, higher packing density creates more adsorption sites, allowing more water molecules to be absorbed, reducing specific humidity in the air, and thereby increasing the change in specific humidity. However, higher-density desiccants have fewer accessible adsorption sites due to tightly packed particles, which reduces their moisture absorption efficiency and limits changes in specific humidity. Additionally, smaller void spaces between particles restrict airflow, slowing the moisture transfer rate. Once the desiccant reaches its saturation point, its ability to further reduce moisture content diminishes, further constraining the change in specific humidity.

In a two-stage dehumidification process, the desiccant gradually becomes more saturated, reducing its ability to absorb moisture and leading to smaller decrease in specific humidity in subsequent stage. Additionally, heat released during exothermic adsorption process raises air temperature, further hindering desiccant’s moisture removal capacity. Consequently, change in specific humidity diminishes further in second stage. For a density value of 3290 kg/m^3^, there is a reduction in change in specific humidity by 33.33%.

#### Moisture removal rate

**Fig. 7:**
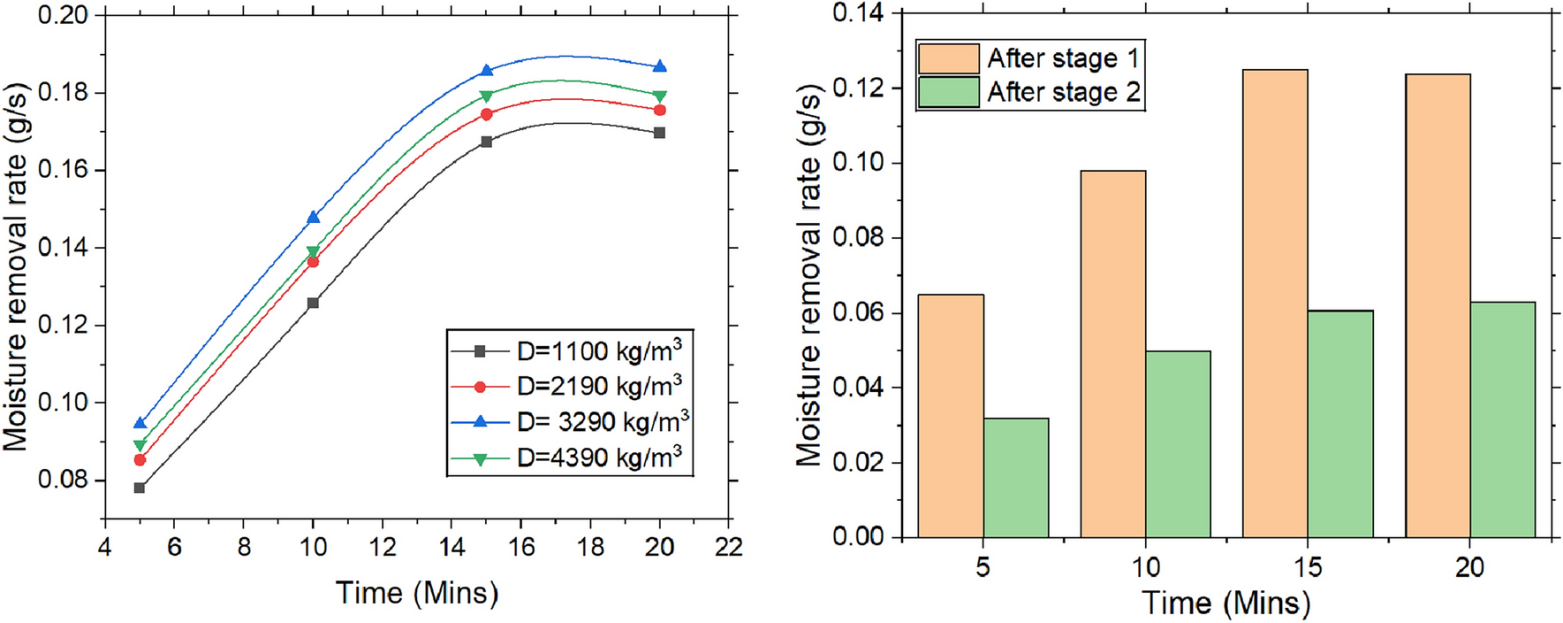
Change in moisture removal rate with the varying time, packing density and the stages.

Figure 7 depicts the variation in moisture removal rate (MRR) over time, packing density, and the number of stages. It is determined by the product of the rate of air flow and the change in specific humidity. Initially, the MRR increases over time until it stabilizes at a steady state. This is because, during desiccant dehumidification, moisture removal is most effective before the desiccant reaches saturation, as more adsorption sites are available. The MRR doubles when the experimental time is extended from 0 to 15 min. Beyond 15 min, the MRR curve levels off, signifying that the moisture removal rate becomes constant. As time progresses, the desiccant reaches thermal equilibrium, where the heat generated by adsorption is balanced by heat dissipating into the environment.

Higher packing density enhances air distribution within the desiccant bed, increasing moisture absorption by providing more active adsorption sites and surface area. This results in a higher MRR and improved dehumidification efficiency. As packing density increases, the desiccant adsorbs moisture more quickly, reaching saturation sooner. Additionally, higher packing density shortens the pathways for water vapor diffusion, speeding up the adsorption process. The closer proximity between water vapor and desiccant particles further boosts dehumidification. However, during the initial phase, the MRR rises rapidly due to more efficient moisture capture, but once a packing density of 3290 kg/m3 is reached, the MRR decreases by 5.55%. As air moves through multiple desiccant stages, the moisture gradient between desiccant surface & air gradually decreases. Initially, high concentration gradient facilitates efficient diffusion of water molecules from air to desiccant. But as air’s moisture content drops and the desiccant nears saturation, diffusion slows, reducing the moisture removal rate with each additional stage. Furthermore, as desiccant approaches saturation, its thermodynamic efficiency for moisture adsorption reduces, making the dehumidification process less effective. With more stages, the desiccant’s ability to further reduce the air’s moisture content diminishes.

#### Dehumidification efficiency

**Fig. 8:**
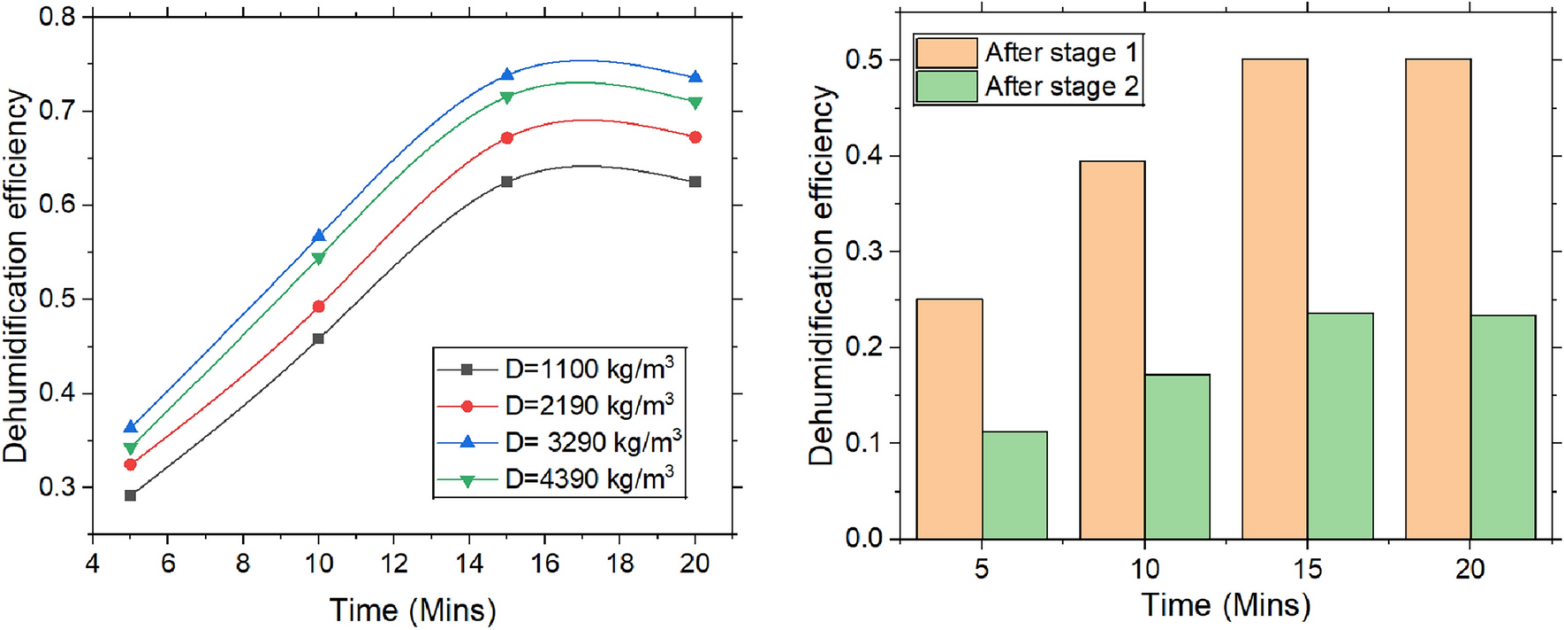
Dehumidification efficiency with the varying time, packing density and the stages.

Dehumidification efficiency as shown in the Fig. 8 is given by the ratio of the difference in specific humidity between inlet & outlet air after second stage, relative to inlet specific humidity and the equilibrium specific humidity. As the desiccant adsorbs water molecules, latent heat is released, causing the desiccant’s temperature to rise. Thermal equilibrium occurs when the heat generated by adsorption is balanced by the heat dissipated to the surrounding environment. The desiccant’s temperature plays a vital role in maintaining its moisture-adsorbing capacity. At higher temperatures, the desiccant sustains a favourable moisture gradient, enhancing dehumidification efficiency. However, as desiccant becomes more saturated, adsorption rate decreases, and system reaches thermal and moisture equilibrium, leading to stabilized dehumidification efficiency. Moreover, as the desiccant saturates, airflow resistance within the packing bed increases, obstructing the air’s movement. This reduces contact time between air & desiccant, limiting moisture removal & contributing to the stabilization of dehumidification efficiency. In desiccant dehumidification systems, an increase in packing density optimizes the distribution of airflow, as fluid flow analysis reveals a more uniform passage of air through the desiccant bed. This consistent exposure to the desiccant material significantly improves moisture removal efficiency. Fluid flow analysis also identifies regions of inadequate airflow or insufficient desiccant interaction, allowing for precise adjustments that enhance system performance. The higher packing density reduces the diffusion paths for water vapor, thereby accelerating the adsorption process and improving mass transfer, which in turn enhances dehumidification efficiency. However, this increased density also leads to higher airflow resistance and a greater pressure drop across the desiccant bed, which may reduce airflow rates. Fluid flow analysis facilitates the balancing of these factors, optimizing packing density to maximize moisture removal while minimizing airflow losses, thereby ensuring the system operates at peak efficiency. As more stages are added, the air faces increased resistance and pressure drop across the desiccant bed, which further restricts airflow and decreases the system’s overall efficiency. The combined effects of diminished adsorption capacity and higher flow resistance lead to a gradual reduction in dehumidification efficiency as the number of stages increases. System gave a maximum dehumidification efficiency of 73.85% & dehumidification efficiency dropped by 2.73% when the packing density increased from 3290 to 4390 kg/m^3^.

#### Coefficient of performance

**Fig. 9:**
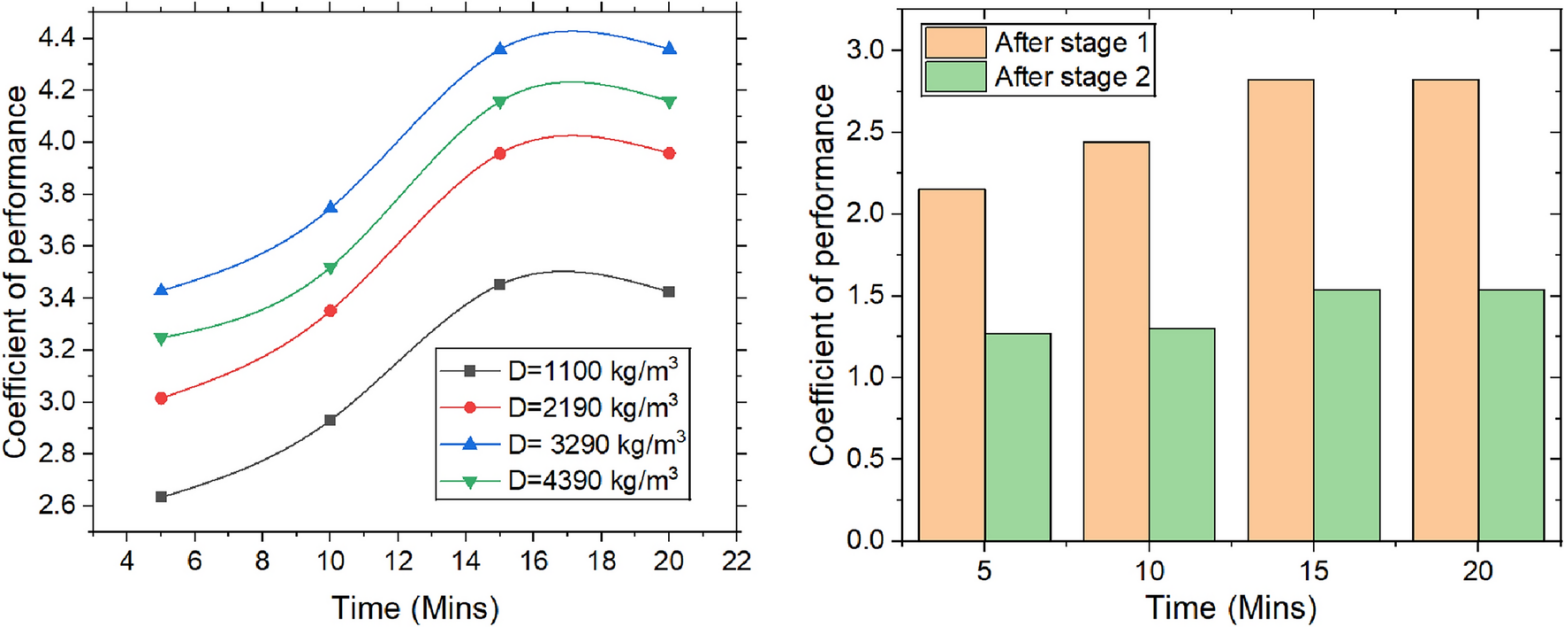
COP with varying time, packing density and the stages.

The COP is defined as the ratio of heating effect to work done by blower, which is maintained at a constant level. Figure 9 illustrates the variation of COP with respect to time, packing density, and the number of stages. The heating effect is determined by the product of the change in enthalpy and the air flow rate. As air passes through the rice desiccant packing, moisture is adsorbed by desiccant, releasing latent heat due to exothermic nature of adsorption process. Over time, this accumulation of heat creates a temperature gradient within the desiccant, resulting in an increase in the temperature of air exiting system and an increase in enthalpy. With a fixed blower power, the COP continues to improve. However, as the desiccant approaches saturation, the rise in enthalpy levels off, and the system reaches thermal equilibrium, where the heat generated by adsorption is balanced by the heat dissipated to the surroundings, stabilizing the heating effect.

At the molecular level, an increased packing density shortens the diffusion path for water vapor molecules, thereby accelerating the exothermic adsorption process and enhancing moisture removal. This results in a greater release of latent heat, which improves the system’s heating effect and increases the coefficient of performance (COP). Additionally, higher packing density promotes more uniform airflow, optimizing the molecular interactions between water vapor and the desiccant, further boosting the COP. Over time, from 0 to 15 min, the COP increases from 3.42 to 4.35 at a packing density of 3290 kg/m^3^, after which it stabilizes. However, when the packing density increases from 3290 kg/m^3^ to 4390 kg/m^3^, the COP decreases by 4.19%. As air moves through each stage of the desiccant bed, moisture is progressively removed, causing the change in specific humidity (or enthalpy) between inlet & outlet air to decrease. Initially, the air contains more moisture, allowing the desiccant to absorb more water and resulting in a larger change in enthalpy. As the air becomes drier in subsequent stages, the amount of moisture available for adsorption diminishes, leading to a smaller change in enthalpy.

#### Mass transfer coefficient

**Fig. 10:**
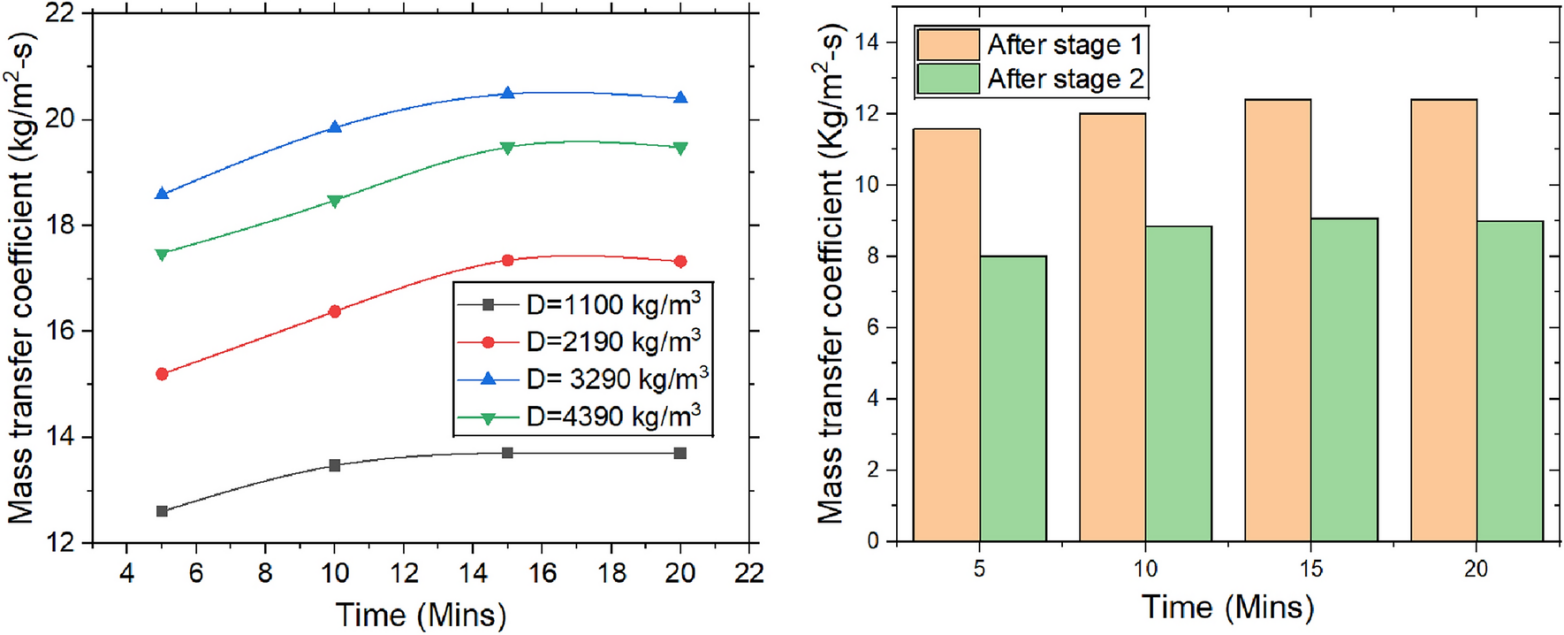
Change in mass transfer coefficient with the varying time, packing density and the stages.

The MTC is defined as the ratio of moisture removal rate to product of the packing surface area & difference between the average & equilibrium specific humidities. It is given by the Fig. 10. In rice desiccant systems, temperature and pressure conditions change over time. As moisture is adsorbed, the temperature of desiccant material rises slightly, with heat being released externally. This rise in temperature boosts the vapor pressure of water, thereby facilitating the transfer of moisture from air to desiccant. Over time, these thermal and pressure changes can optimize mass transfer process. Additionally, interaction between water vapor molecules & desiccant particles becomes more pronounced, fostering more efficient mass transfer. This interaction may result in the formation of a thin liquid film of adsorbed water on the surface, which further enhances the transfer of water molecules into the desiccant material. As the moisture removal rate (MRR) is present in the numerator, any rise in the MRR directly leads to an increase in mass transfer coefficient. After 15 min, the rice packing bed reaches an equilibrium moisture removal rate. At this point, various system parameters, such as airflow, temperature, and humidity, also stabilize. The system naturally adjusts to a steady state, where moisture removal occurs at a constant rate, and the mass transfer coefficient reflects this stable operating condition.

As packing density increases from 1100 to 3290 kg/m^3^, the surface area available for moisture adsorption expands, providing more active sites and shortening diffusion distances, which accelerates moisture transfer and increases the mass transfer coefficient by 49.38%. However, when the density increases further from 3290 to 4390 kg/m^3^, the coefficient drops by 4.88%. Higher packing density also reduces porosity, increasing airflow resistance and the risk of channelling, which can disrupt airflow uniformity and efficiency. Coupled with a higher pressure drop, these factors may ultimately limit the mass transfer coefficient at very high densities, despite the initial benefits of increased surface area and shorter diffusion paths. In the early stages of dehumidification, the air contains a high concentration of water vapor, creating a steep concentration gradient between the air and the desiccant. This large gradient facilitates rapid diffusion of water vapor molecules from air to desiccant material. The greater difference in specific humidity, the faster the molecular movement of water vapor, resulting in a higher mass transfer coefficient. However, as the air becomes progressively drier in subsequent stages, the concentration gradient decreases, reducing the driving force for molecular diffusion. This slows the movement of water vapor molecules, leading to a decline in the mass transfer coefficient. Additionally, as the desiccant approaches saturation, the adsorption rate decreases because water vapor molecules have lower kinetic energy due to the reduced vapor pressure difference. This thermodynamic shift further limits molecular movement and moisture transfer, contributing to a lower mass transfer coefficient.

#### Latent and sensible heat ratio

**Fig. 11:**
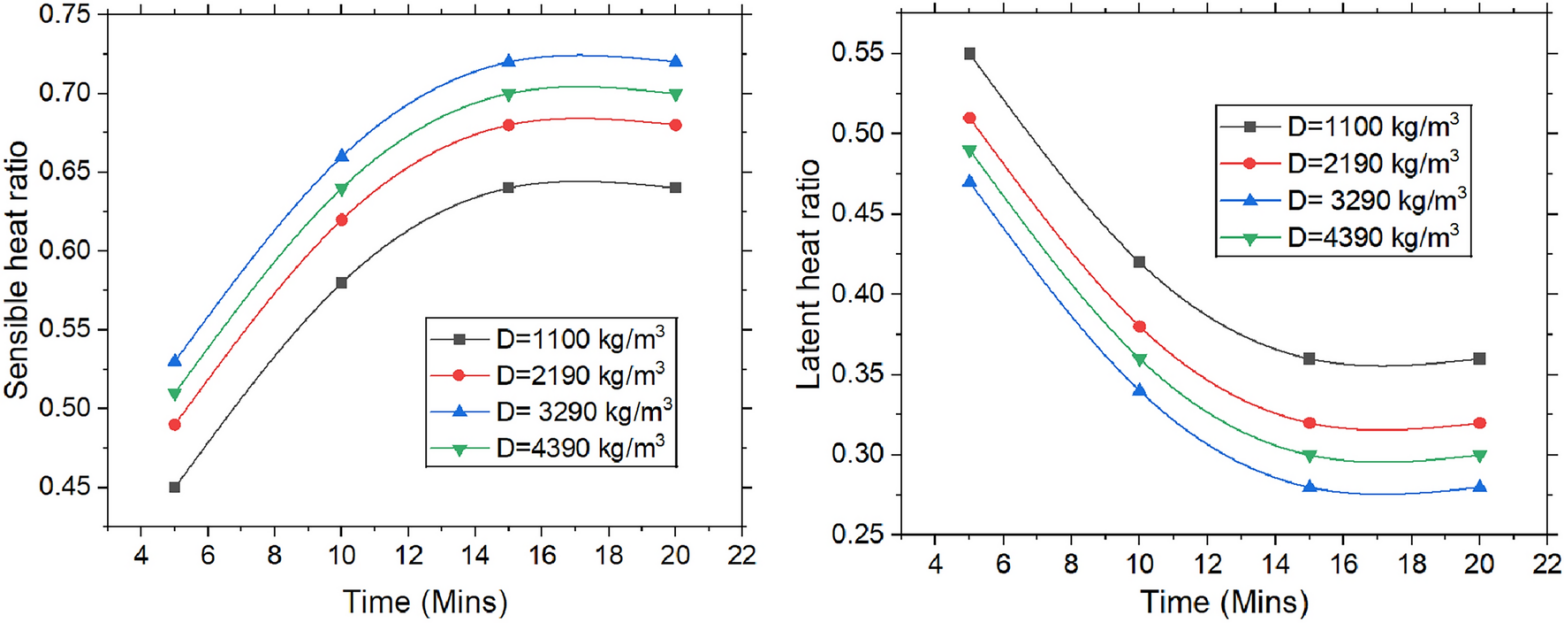
Change in LHR and SHR with the varying time, packing density and the stages.

Figure 11 illustrates the variation of both latent heat ratio & sensible heat ratio over time. The latent heat ratio is defined as total latent heat required to adsorb moisture, divided by the total heat transfer rate. Similarly sensible heat is given by the sensible heat taken from air and desiccant during dehumidification to overall heat transfer rate. Initially, when the desiccant is exposed to the air, it absorbs both sensible heat (which is related to temperature) and latent heat (which is associated with moisture content). As moisture is removed from the air, the temperature may drop, leading to an increase in the latent heat ratio. However, as the dehumidification process progresses and the desiccant becomes saturated, its capacity to adsorb extra moisture decreases. At this point, the cooling effect of desiccant weakens, and the air temperature begins to rise. As the focus shifts toward primarily removing moisture (latent heat) rather than cooling the air, the sensible heat ratio increases. This occurs because the sensible heat ratio reflects the proportion of sensible heat (temperature-related heat) relative to the total heat transfer rate. As moisture removal becomes the dominant process, the contribution of sensible heat grows, causing the sensible heat ratio to rise over time. As the packing density of the desiccant increases, more material is available to absorb moisture, allowing the dehumidifier to reduce humidity more quickly. This leads to a higher sensible heat ratio, as the system’s heat absorption focuses more on temperature-related heat. Meanwhile, as the desiccant becomes saturated, its ability to absorb latent heat diminishes, causing the latent heat ratio to decrease. Thus, higher packing density results in increased sensible heat ratio and reduced latent heat ratio. Density of 3290 kg/m^3^ yielded a higher sensible heat ratio of 0.72 whereas the lower density 1100 kg/m^3^ yielded a higher latent heat ratio of 0.55.

#### Velocity of air and pressure drop

**Fig. 12:**
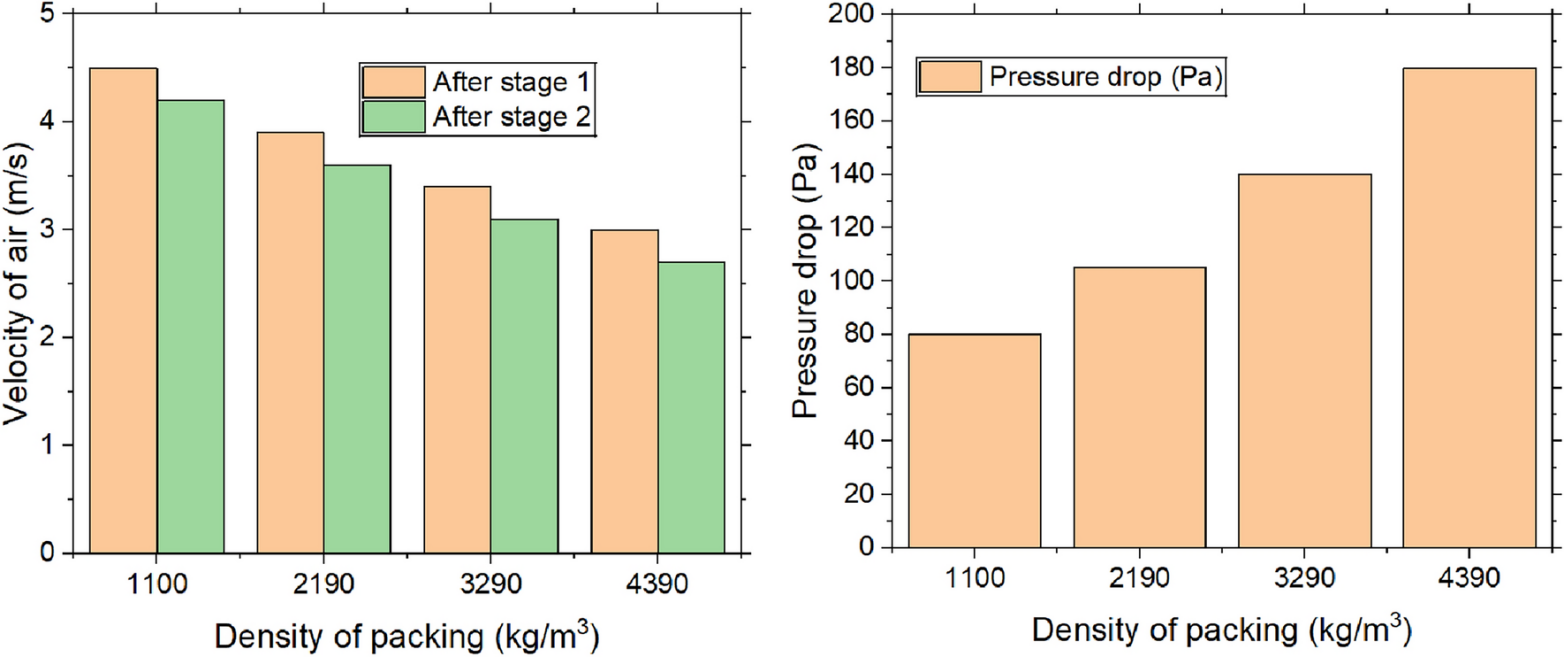
Velocity of air and pressure drop with the density of packing.

Figure 12 delineates the velocity distribution at varying packing densities within stages 1 and 2, along with the corresponding pressure drop across the packing at both the inlet and outlet. As density of desiccant packing increases, velocity of air diminishes due to the augmented resistance to airflow. A higher packing density introduces more desiccant material, such as pellets, which obstructs the air’s path. This increased material density generates greater friction and resistance, disrupting the smooth flow of air through the dehumidifier. This effect can be likened to an increase in the “drag” exerted on the air, thereby reducing its velocity. Consequently, the lower air velocity enhances the interaction time between air & desiccant, which can potentially improve dehumidification process. Within each desiccant packing stage, air molecules continually collide with the desiccant material, further contributing to drag and slowing the airflow. Additionally, as packing density rises, the number of obstacles encountered by the air increases, intensifying the resistance. This reduction in air velocity arises from the combined effects of friction, turbulence, and the increased surface area the air must traverse, resulting in a gradual decline in speed as it progresses through the packing stages. Nevertheless, achieving the optimal balance between air velocity and moisture absorption efficiency is crucial in the design of the dehumidifier system to ensure optimal performance.

**Fig. 13:**
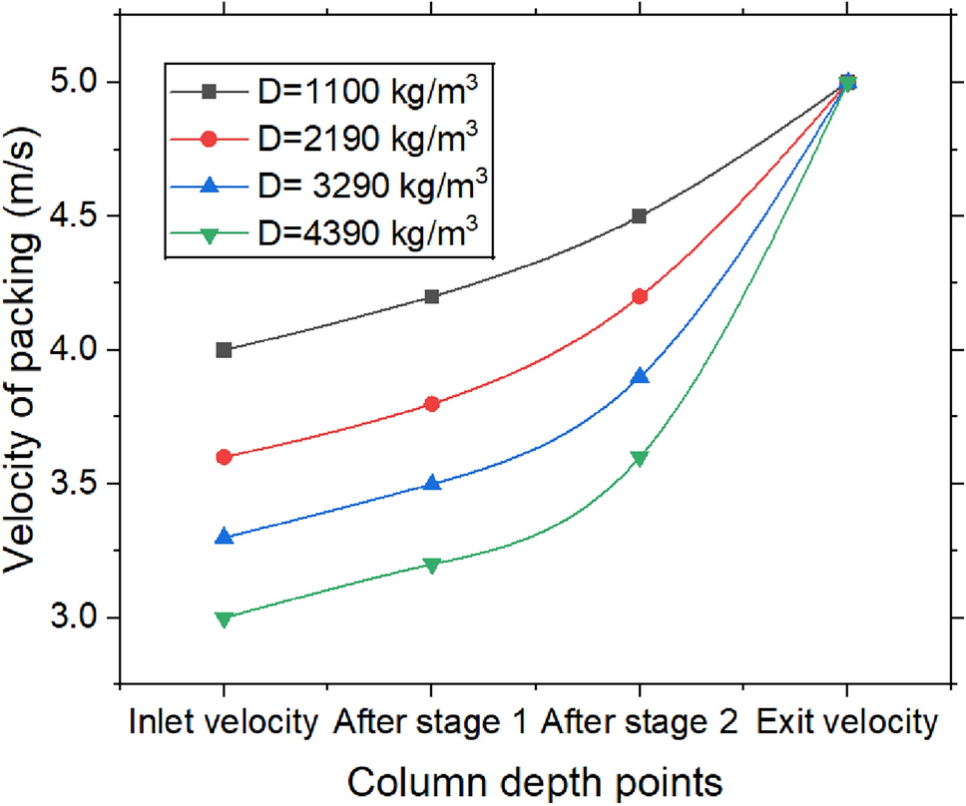
Change in velocity of air and pressure drop with the density of packing (Top to bottom section of the dehumidifier column).

As per Fig. 12 the heightened interaction between air & desiccant material engenders increased flow resistance, which subsequently results in a greater pressure drop. In accordance with Darcy’s law for flow through porous media, pressure drop is directly proportional to flow resistance, which escalates with packing density. As the packing density increases, the air must exert more force to overcome the friction and turbulence generated by the desiccant particles. Thus, with higher packing density, the pressure necessary to sustain airflow through the system also rises, culminating in a more significant pressure drop across the desiccant packing. The pressure drop across packing is found to be 180 Pa. Figure 13 illustrates the variation in air velocity along the duct column from top to bottom. A blower, positioned at the bottom of the column, creates a suction effect with a velocity of 5 m/s. The graphs show a rise in air velocity from the top to the bottom section. Since the blower is located at the bottom, air velocity is greater in the lower section. In contrast, the velocity is lower in the top and intermediate sections due to the desiccant packing, which causes drag, friction, and turbulence as the air interacts with the desiccant particles. At the top section, the air encounters more particles, increasing the surface area for interaction, which further amplifies the resistance to airflow. This heightened resistance leads to a reduction in air velocity as it moves upward, while the velocity increases toward the bottom. Air velocity is found to be higher in sections with low-density packing compared to those with high-density packing. High-density packing, consisting of more particles or smaller voids, increases the surface area in contact with the air. As a result, the air molecules collide more frequently with the desiccant material, generating greater friction and turbulence, which slows down the airflow and reduces air velocity. The velocity change from top to bottom for densities of 1100 kg/m^3^, 2190 kg/m^3^, 3290 kg/m^3^, and 4390 kg/m^3^ are observed to be 25%, 38.88%, 51.51%, and 66.66%, respectively.

After conducting the experiments, the maximum performance parameters are presented stage-wise in the table. It was found that a packing density of 3290 kg/m^3^ and an operating time of 15 min yielded the highest results. Table 3 displays the optimal performance parameters from the dehumidification experiments using rice as the desiccant.

**Table 3:** Maximum results obtained after the experimentation.

Sl. no	Performance parameters	Stage 1	Stage 2	Overall performance parameters
1	Chane in temperature (ºC)	2.3	1.3	3.6
2	Change in specific humidity (g/kg)	1.2	0.8	2
3	Moisture removal rate (g/s)	0.12	0.06	0.18
4	Dehumidification efficiency (%)	0.50	0.23	0.73
5	Coefficient of performance	2.82	1.53	4.35
6	Mass transfer coefficient (kg/m^2^-s)	12.4	9.08	20.48
7	Sensible heat ratio	0.5	0.22	0.72
8	Latent heat ratio	0.18	0.10	0.28
9	Maximum Pressure drop (pa)	–	–	180
10	Velocity of air (m/s) Initial velocity = 5 m/s	3.4	3.1	–
11	Optimum packing density (kg/m^3^)	–	–	3290
12	Optimum time (mins) to achieve steady state condition	–	–	15

The current system, which utilizes rice as a desiccant, operates for 4 h daily over a four-week period before being replaced. During long-term use, the performance of rice-based desiccant packing material may undergo notable changes due to environmental exposure and repeated moisture absorption cycles. One key concern is the structural stability of the packing; over time, rice particles may break down, compact, or disintegrate, reducing airflow and increasing pressure drop across the desiccant chamber. Additionally, prolonged exposure to humidity and temperature variations can lead to microbial growth or spoilage, further compromising the material’s integrity. Dehumidification performance may also degrade as the rice loses its moisture absorption capacity due to saturation or irreversible chemical and physical changes. These factors highlight the importance of evaluating both the mechanical durability and moisture-regaining ability of rice desiccant over extended operational periods to ensure consistent and reliable performance.

### Thermal comfort

Table 4 presents the values for DBT & RH across varying rice packing densities and experimental durations. The American Society of Heating, Refrigerating and Air-Conditioning Engineers (ASHRAE) has built standards for thermal comfort, providing essential guidelines to ensure that indoor environments are conducive to the well-being of individuals. Temperature & relative humidity are among most influential factors in thermal comfort. ASHRAE Standard 55, "Thermal Environmental Conditions for Human Occupancy," outlines the recommended conditions for thermal comfort across a variety of indoor settings. ASHRAE advises a comfortable temperature range of 22 to 27 °C for typical indoor environments, although this range may adjust based on activity level and attire^29^. Additionally, the ideal relative humidity (RH) for optimal comfort falls between 40 and 60%**.** The values obtained for varying packing densities and experimental times are within the acceptable thermal comfort standards, indicating that the outlet air can be utilized effectively within the defined space.

**Table 4:** Temperature and relative humidity with the density and time at the outlet to determine the thermal comfort.

Thermal comfort condition: temperature: 22–27ºC and RH: 40–60% (ASHRAE standards)				
	5 min	10 min	15 min	20 min
D = 1100 kg/m^3^	25.6 ºC	26.4 ºC	27 ºC	27 ºC
D = 2190 kg/m^3^	25.9 ºC	26.7 ºC	27.3ºC	27.3 ºC
D = 3290 kg/m^3^	26.1 ºC	26.9 ºC	27.6 ºC	27.6 ºC
D = 4390 kg/m^3^	26 ºC	26.8 ºC	27.5 ºC	27.5 ºC
D = 1100 kg/m^3^	54%	52%	51%	50%
D = 2190 kg/m^3^	52%	51%	51%	49%
D = 3290 kg/m^3^	53%	51%	50%	48%
D = 4390 kg/m^3^	50%	49%	48%	46%

#### Psychrometric graph representing the state of air in stage 1 and stage 2

**Fig. 14:**
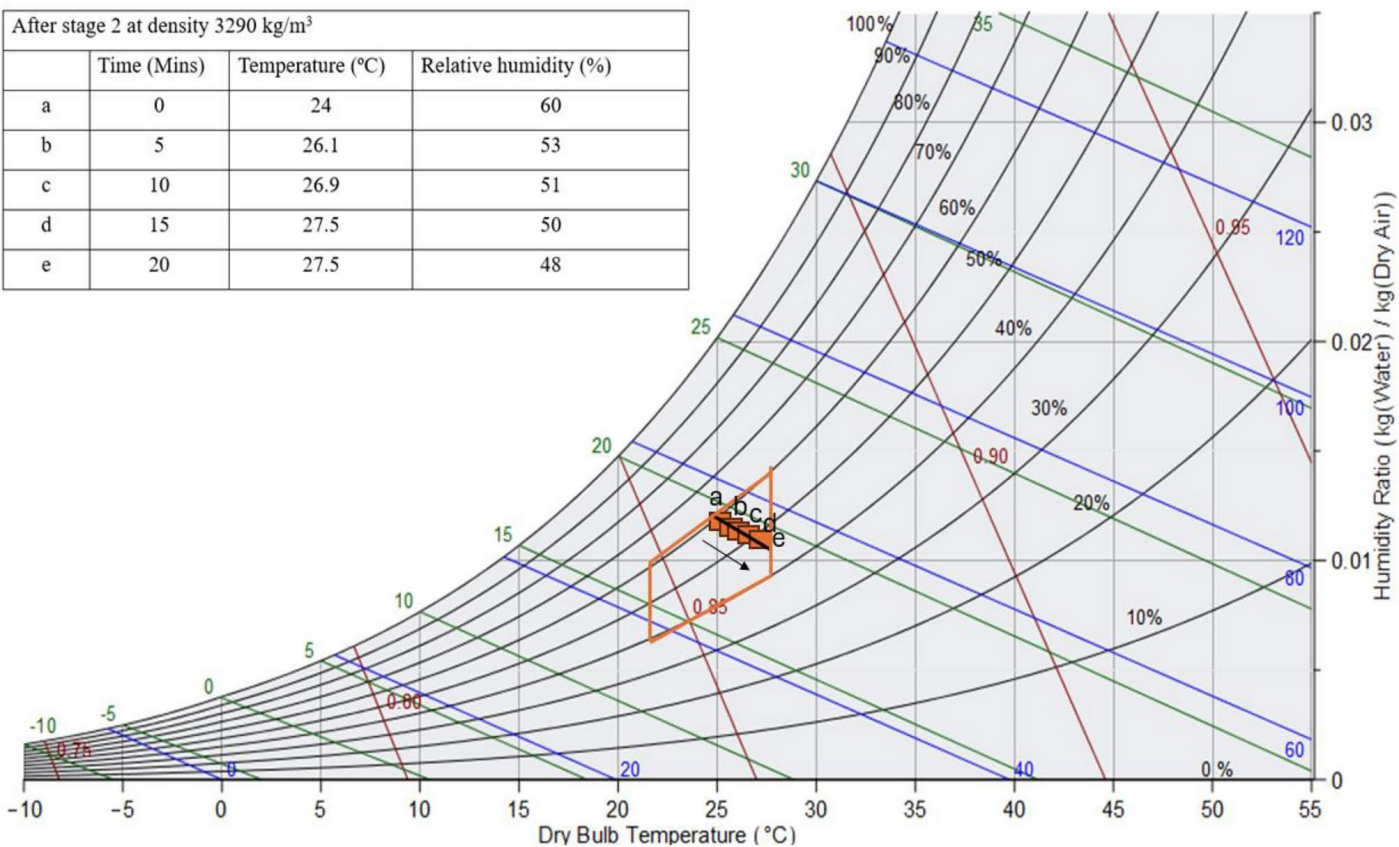
Psychrometric graph to determine the characteristics of exiting air.

Thermal comfort is essential for health, well-being, and productivity. Extreme temperatures can lead to discomfort, stress, and health problems, while maintaining an optimal environment enhances focus, efficiency, and performance. By optimizing heating and cooling systems to achieve thermal comfort, energy consumption is reduced, supporting sustainable design. Architects incorporate thermal comfort into building systems to improve user satisfaction and reduce operational costs. Additionally, it has a positive impact on mood and cognitive function, fostering a more relaxed atmosphere. Figure 14 presents the psychrometric chart, showing variation in DBT and specific humidity. Based on experimental data, the RH and DBT of air passing through a packing density of 3290 kg/m^3^ after stage 2 are plotted. The temperature of desiccant rises & specific humidity drops. The thermal comfort envelope, according to ASHRAE standards, is also shown and the data indicates that exiting air is within thermal comfort limit.

## Validation of current results with literature

Table 5 presents a comparison between the current results and those from other dehumidifier units. Two performance parameters, namely MRR & dehumidification efficiency, are considered. The findings indicate that the experimental results align closely with the values reported in the literature.

**Table 5:** Comparison between present results with literature values.

Sl. no	Name of the author	Type of desiccant used	Inlet parameters	Performance parameters
1	Present study	Rice solid desiccant	Time variation:0–20 min Density variation: 1100 to 4390 kg/m^3^ Number of stages:2	MRR = 0.18 g/s, COP = 4.35 & Dehumidification efficiency = 73%
2	Naik et al.^20^	Two stage dehumidifiers: Lithium Chloride (Li Cl) & Silica gel (Liquid and Solid desiccant)	Air flow rate = 0.8 to1.7 kg/s	MRR = 0.15 g/s & Dehumidification efficiency = 68%
3	Kamsah et al.^30^	Silica del desiccant with the Honeycomb configuration	Ait temperature at the inlet is 40-60ºC and the velocity of air is at 6 to 10 m/s	MRR = 0.17 g/s and Dehumidification efficiency = 60%
4	Kumar et al.^31^	Calcium Chloride (Ca Cl_2_)	Inlet temperature of 30 to 42ºC Inlet RH = 75 to 90%	Moisture removal rate of 5 g/s & dehumidification efficiency of 78%
5	Seenivasan et al.^32^	Li Cl	Temperature of desiccant = 25ºC and RH = 70%	MRR of 0.8 g/s & dehumidification efficiency of 68%

## Conclusions

Dehumidification experiments are conducted with variation of time, density & number of stages. The following conclusions are drawn and presented.

1. The majority of heat is generated in the first stage, leading to a temperature rise of 2.3 °C, which then drops to 1.3 °C. Over the course of 0 to 15 min, the temperature change increased by 63.63%. Moisture adsorption increased with packing density up to 3290 kg/m^3^, after which the adsorption capacity dropped significantly by 33.33%.
2. The MRR is proportional to change in specific humidity, reaching a steady state over time. The maximum MRR is found to be 0.18 g/s.
3. The system achieved the highest dehumidification efficiency of 73.85%, which decreased by 2.73% when the packing density increased from 3290 to 4390 kg/m^3^. Over a period of 0 to 15 min, the COP increased from 3.42 to 4.35 at a packing density of 3290 kg/m^3^, after which it stabilized.
4. The maximum mass transfer coefficient is 20.40 kg/m^2^·s at a packing density of 3290 kg/m^3^. When the density increases further from 3290 to 4390 kg/m^3^, the coefficient decreases by 4.88%.
5. An increase in packing density elevates the sensible heat ratio while diminishing the latent heat ratio. At a density of 3290 kg/m^3^, the sensible heat ratio reaches 0.72, whereas a lower density of 1100 kg/m^3^ leads to a higher latent heat ratio of 0.55.
6. The velocity change from top to bottom for densities of 1100 kg/m3, 2190 kg/m^3^, 3290 kg/m^3^, and 4390 kg/m^3^ is observed to be 25%, 38.88%, 51.51%, and 66.66%, respectively. The maximum pressure drops across the packings is found to be 180 Pa at the highest density.
7. The values obtained for different packing densities and experimental durations fall within the acceptable thermal comfort range defined by ASHRAE standards, suggesting that the outlet air can be effectively used within the designated space.

The dehumidification experiments using rice as a desiccant revealed numerous benefits, including cost-effectiveness, natural abundance, and a high moisture adsorption capacity. Additionally, rice proved effective in preventing Mold, is non-toxic, safe to handle, reusable, biodegradable, easy to transport, zero carryover and more affordable than many alternative desiccants. These qualities position rice as a versatile and environmentally friendly solution for moisture control in desiccant dehumidifiers. As a sustainable system, it makes a meaningful contribution to achieve SDG 7 (Affordable & Clean Energy) & SDG 11 (Sustainable Cities & Communities).

### Limitations of the study

Rice as a desiccant has several limitations that reduce its effectiveness. Its moisture absorption is highly dependent on temperature and humidity, with performance fluctuating significantly in extreme conditions. Furthermore, when rice becomes saturated and is not properly dried, it can foster Mold and bacterial growth, posing health risks and causing unpleasant odors. Compared to specialized desiccants, rice has a short lifespan and needs to be replaced once it reaches its moisture capacity. Achieving significant moisture removal requires a large quantity of rice, making it impractical for larger spaces due to its bulk. Additionally, unlike silica gel, rice cannot be regenerated for reuse and must be entirely replaced once saturated. The study’s limitations are addressed by adjusting air velocity and packing density to optimize performance. Additionally, the rice desiccant is replaced every four weeks with fresh material to enhance the efficiency of the dehumidification process.

### Practical applications

1. The rice-type vertical axis two-stage dehumidifier is widely used in post-harvest agricultural processes, particularly for drying rice to safe moisture levels for storage and milling.
2. This method is crucial in regions with high humidity, where traditional sun drying is unreliable or insufficient.
3. It is used in pharmaceutical industries to maintain moisture inside the space.
4. Used in paper industries to maintain dry air.

## Data Availability

The data related to the current research will be made available to the Journal upon request. Data that supports the findings have been deposited in the Mandeley Repository: 10.17632/x9zzfb7n6s.1. https://data.mendeley.com/datasets/x9zzfb7n6s/1.

## Abbreviations

COP: Coefficient of performance
DBT: Dry bulb temperature, °C
DCHE: Desiccant-coated heat exchangers
HDS: Hybrid dehumidification system
HE: Heating effect, W
MCHE: MOF-coated heat exchanger
MOF: Metal–organic frameworks
MRR: Moisture removal rate, g/s
RH: Relative humidity, %
RSS: Root sum of squares
SCHE: Silica gel-coated heat exchangers
SMER: Specific moisture extraction rate, kg/kW-hr
VCR: Vapor compression refrigeration

## Variables

T: Temperature, ºC
W_1_: Inlet specific humidity, g/kg
W_2_: Specific humidity after stage 1, g/kg
W_3_: Specific humidity after stage 2 or outlet, g/kg
h_1_: Specific enthalpy at inlet, g/kg
h_2_: Specific enthalpy after stage 1, g/kg
h_3_: Specific enthalpy after stage 2 or outlet, g/kg
$\dot{W}$: Total power consumed, W
W_eq_: Equilibrium specific humidity, g/kg
W_av_: Average specific humidity, g/kg
ΔRH: Change in relative humidity %
$\dot{{m}_{a}}$: Air flow rate (kg/s)
$$\dot{{m}_{w}}$$: Condensation rate g/s
$\dot{Q}$: Effect of heating, W
${\dot{W}}_{B}$: Power consumed by blower, W
C_p_: Specific heat at constant pressure, J/kg˚C
$\dot{W}$: Total power consumed, W
X: Independent variable
Y: Uncertainty intervals
G: Uncertainty function
Y_G_: Total Uncertainty
$${\eta }_{d}$$: Dehumidification efficiency, %
A: Area, m^2^
ΔW: Specific humidity difference, g/kg
K: Coefficient of mass transfer, kg/m^2^-s
ρ: Density, kg/m^3^
g: Acceleration due to gravity, m/s^2^
Z: Height of the fluid in the manometer, m
$\frac{\partial (\dot{{m}_{W}})}{\dot{{m}_{W}}}$: Uncertainty in the moisture removal rate
$\frac{\partial (COP)}{COP}$: Uncertainty in the coefficient of performance
$\frac{\partial ({\eta }_{d})}{{\eta }_{d}}$: Uncertainty in the dehumidification efficiency
$\frac{\partial (K)}{K}$: Uncertainty in the mass transfer coefficient

## Subscripts

1: Inlet
2: After stage 1
3: After stage 2
av: Average
eq: Equilibrium
a: Air
w: Water
B: Blower
G: Uncertainty

## Chemicals

C_3_H_3_AlO_6_: Aluminium fumarate
Li Cl: Lithium chloride
Li Br: Lithium bromide
MgCl_2_: Magnesium chloride
CaCl_2_: Calcium chloride
Si O_2_: Silica gel
